# Impact of baseline visual acuity, time-in-range and early treatment on functional outcomes in DMO: insights from the IRISS outcomes

**DOI:** 10.1038/s41433-025-04102-8

**Published:** 2025-12-03

**Authors:** Igor Kozak, Ramin Khoramnia, Simon R. Taylor, Usha Chakravarthy

**Affiliations:** 1 Department of Ophthalmology and Vision Science, University of Arizona, Tucson, AZ USA; 2 University Eye Clinic Heidelberg, Heidelberg, Germany; 3 Department of Ophthalmology, University Hospital Carl Gustav Carus, TU Dresden, Germany; 4 Department of Ophthalmology, University of Surrey, Guildford, UK; 5 Queen’s University Belfast, Belfast, UK; 6 NIHR Moorfields Clinical Research Facility, London, UK; 7 The Queen’s University and Royal Group of Hospitals Trust, Belfast, Ireland; 8 Gloucestershire Hospitals NHS, Cheltenham, England; 9 Unit Frimley Park Hospital, Surrey, England; 10 Heart of England NHS Foundation Trust, Birmingham, England; 11 Laser and Retinal Research Unit, King’s Health Partners, London, UK; 12 Royal Surrey County Hospital, Guildford, England; 13 ICORG - Imperial College, London, UK; 14 Manchester Royal Eye Hospital, Manchester, England; 15 Derriford Hospital, Plymouth, Devon, England; 16 Royal Hallamshire Hospital, Sheffield Teaching Hospitals NHS, Sheffield, England; 17 Sandwell and West Birmingham Hospital NHS Trust, Birmingham, England; 18 Queen Elizabeth Hospital, Birmingham, England; 19 James Paget University Hospital, Gorleston, England; 20 Sunderland Royal Hospital, Sunderland, UK; 21 Wrightington, Wigan and Leigh Eye Unit, WWL NHS Trust, Wigan, England; 22 University Hospital Leicester NHS, Leicester, England; 23 Leighton Hospital, Crewe, England; 24 Kent and Canterbury Hospital, East Kent Hospitals University, Canterbury, England; 25 Queen Alexandra Hospital, Portsmouth Hospitals NHS, Portsmouth, UK; 26 Royal Free Hospital, London, UK; 27 New Cross Hospital, The Royal Wolverhampton NHS, Wolverhampton, England; 28 Calderdale Hospital Eye Clinic, Calderdale and Huddersfield NHS Trust, Huddersfield, England; 29 Aintree University Hospital, Liverpool, England; 30 Bristol Eye Hospital, University Hospitals Bristol, Bristol, England; 31 Hull & East Yorkshire Eye Hospital, Hull, England; 32 Queen’s Hospital, Burton Hospitals NHS, Burton-on-Trent, England; 33 Maidstone Hospital, Maidstone, Kent, England; 34 Royal Derby Hospital, Derby, England; 35 Whipps Cross University Hospital, London, UK; 36 Norfolk and Norwich University Hospital, Norwich, England; 37 University Medical Center, Johannes Gutenberg-University Mainz, Mainz, Germany; 38 University Hospital Tuebingen, Tuebingen, Germany; 39 Department of Ophthalmology, University of Bonn, Bonn, Germany; 40 University Medical Center Hamburg-Eppendorf, Hamburg, Germany; 41 University Eye Hospital Leipzig, Leipzig, Germany; 42 University of Düsseldorf, Düsseldorf, Germany; 43 Eye Centre Spreebogen, Berlin, Germany; 44 International Vision Correction Research Centre, University of Heidelberg, Heidelberg, Germany; 45 Gemeinschaftspraxis am Glacis, Torgau, Germany; 46 Universitätsklinikum Frankfurt, Klinik für Augenheilkunde, Frankfurt, Germany; 47 Krankenhaus Dresden-Friedrichstadt, Dresden, Germany; 48 Universitätsklinikum Carl Gustav Carus, Klinik für Augenheilkunde, Dresden, Germany; 49 Universitätsklinikum des Saarlandes, Augenklinik, Homburg, Germany; 50 AIBILI, Coimbra, Portugal; 51 Department of Ophthalmology, Porto Medical School / Hospital S. João, Porto, Portugal; 52 Instituto de Retina e Diabetes Ocular de Lisboa (IRL), Lisbon, Portugal; 53 Espaço Médico de Coimbra, Coimbra, Portugal; 54 Hospital de Vila Franca de Xira, Vila Franca de Xira, Portugal; 55 Hospital de Santo António, Porto, Portugal; 56 Centro Hospitalar de Leiria, Leiria, Portugal; 57 Centro Hospitalar de Lisboa Ocidental, EPE, Lisbon, Portugal; 58 Hospital Pedro Hispano (Unidade Local de Sáude de Matosinhos, EPE), Senhora da Hora, Portugal

**Keywords:** Retinal diseases, Outcomes research

## Abstract

**Background/objectives:**

The initial visual acuity (VA) prior to treatment initiation can significantly influence long-term visual outcomes. The current analysis aimed to examine change in VA by baseline vision categories and their effects on time spent within visual change categories in patients with diabetic macular oedema (DMO) who underwent treatment with the intravitreal fluocinolone acetonide (FAc) implant.

**Subjects/methods:**

This was a post-hoc analysis of the IRISS-Registry Data. Time-in-range (TIR) was calculated based on three VA letter-score-thresholds: ≥70, ≥65, and ≥60 ETDRS letters after treatment initiation. TIR was stratified by baseline VA in three groups: 0–33, 34–68, and 69–100 letters. The primary outcome was the mean TIR for the ≥70 letters threshold (equivalent to 6/12 in Snellen).

**Results:**

A total of 671 eyes from 542 patients were included. VA improved significantly in all VA swimlane groups, with 84.8%, 71.7%, and 60.0% of eyes in the 0–33, 34–68, and 69–100 baseline VA categories, respectively, showing maintained or improved VA at 36 months (*p* = 0.0367). The mean TIR for the ≥70 letter threshold was significantly longer in the 69–100 letters subgroup (892.7 ± 413.4 days) compared to the 34–68 (648.4 ± 366.4 days) and 0–33 (251.3 ± 175.9 days) subgroups (*p* < 0.0001). No significant differences in TIR were observed based on the duration of DMO or the number of previous anti-angiogenic injections.

**Conclusions:**

Eyes with better initial VA maintained functionally better visual acuity for longer following FAc implant treatment. TIR emerged as a potentially clinically relevant endpoint for evaluating long-term treatment outcomes in DMO, offering a broader perspective than traditional VA measures.

## Introduction

Diabetic macular oedema (DMO) is a leading cause of visual impairment in diabetic patients, with global incidence rising steadily [1]. Early detection and prompt treatment are critical to reducing vision loss [2]. Intravitreal therapies, including vascular endothelial growth factor (VEGF) inhibitors and sustained-release corticosteroid implants, have revolutionised DMO management [3–5]. Despite advancements, approximately 40% of patients show suboptimal responses due to multifactorial pathogenesis involving inflammatory pathways beyond VEGF [6–8], delayed treatment initiation leading to irreversible retinal damage [9–12], and issues with treatment adherence and regimen efficacy [13–15].

The limited durability of standard therapies has driven interest in alternatives that offer extended action and broader mechanisms. Intravitreal steroid implants, effective for over a decade, provide long-term control of oedema and stabilise vision for up to 36 months, but risks of cataract formation and elevated intraocular pressure (IOP) limit their widespread use [3–5, 16]. The ILUVIEN implant (0.19 mg fluocinolone acetonide, Alimera Sciences Europe Ltd.) mitigates some of these concerns and has demonstrated efficacy in pivotal clinical trials, showing improvements in best-corrected visual acuity (BCVA) and reductions in central retinal thickness (CRT) [16–19]. In addition, data from routine care registries indicated better preservation of visual function when the implant is administered early in the DMO treatment pathway [20, 21].

Given the prolonged nature of DMO management, evaluating treatment efficacy over extended periods is crucial. Time-in-range (TIR), originally used to assess glucose control [22], has emerged as a dynamic measure of visual acuity (VA) stability. TIR represents the duration during which VA remains at or above a clinically relevant threshold, typically 69 ETDRS letters (6/12 vision) [23]. This metric captures visual fluctuations more effectively than fixed timepoint assessments.

Baseline factors significantly influence treatment outcomes, necessitating precise evaluation of therapeutic responses. Optical coherence tomography (OCT) measures of CRT and central subfield thickness (CST) are standard parameters in clinical trials [24, 25].

This post-hoc analysis of the IRISS study aimed to examine VA changes over 36 months post-fluocinolone acetonide (FAc) implant in DMO patients, stratified by baseline VA. Additionally, TIR was assessed across the entire cohort and in subgroups based on initial visual acuity, offering insights into long-term treatment effectiveness and its relationship to baseline characteristics.

This study advances the understanding of the role of sustained-release steroid therapy in chronic DMO management and highlights the importance of comprehensive outcome measures beyond traditional endpoints.

## Methods

### Study design

This post-hoc analysis utilised data from the IRISS study (NCT01998412), a post-approval safety study (PASS) assessing the safety and visual acuity (VA) outcomes of the FAc implant. Conducted across the UK (31 sites), Germany (11 sites), and Portugal (5 sites), the study ran from January 2014 to January 2020. Ethical approval was obtained, and all participants provided informed consent [20, 21]. A total of 562 participants were enrolled, with 556 receiving the FAc implant (*N* = 695 eyes). The maximum follow-up was 1978 days ( ~ 65 months), with the final data extraction on April 28, 2020.

### Study participants

Eligible patients received the 0.19 mg FAc implant for any clinical reason, including chronic DMO. Data collected included VA, cataract onset, intraocular pressure (IOP) changes, and treatments during follow-up.

### Data handling and analysis

Study eyes were subclassified by duration of DMO as follows:

“Short-term DMO”: duration of DMO ≤3.6 years

“Long-term DMO”: duration of DMO >3.6 years

Study eyes were also subclassified according to baseline VA into three groups: ≥0 and ≤33 ETDRS letters; ≥34 and ≤68 ETDRS letters; and, ≥69 and ≤100 ETDRS letters, respectively.

### Time-in-range

Time-in-range (TIR) analysis was conducted to evaluate the duration for which best-corrected visual acuity (BCVA) remained above clinically meaningful thresholds, reflecting the consistency of functional vision over time (see Annex I in supplementary material). Three thresholds were pre-specified: ≥6/12 minus 10 letters, ≥6/12 minus 5 letters, and ≥6/12. For each eye, the TIR was computed as the total number of days during which BCVA was assumed to remain above the threshold, divided by the total follow-up time. BCVA was assumed to remain constant between consecutive recorded visits (last observation carried forward until the next measurement). Sensitivity analyses were conducted using thresholds from 100 to 0 ETDRS letters (Snellen equivalents 20/10 to 20/800), in 1-letter increments. Visual acuity was assessed at baseline and approximately every 3 to 6 months, though exact timing varied slightly between centres. No imputation was applied to missing data; eyes without a baseline VA record were excluded from swimlane allocation and TIR computation.

### Other study outcomes

Mean VA was stratified by swimlane, and the following proportion of eyes achieving a VA (a) improvement of ≥5, ≥10, and ≥15 letters by swimlane, (b) maintaining stable VA (±4 letters) by swimlane, (c) demonstrating either improved or stable VA by swimlane, and (d) experiencing a VA loss of ≥5, ≥10, and ≥15 letters by swimlane. A survival analysis illustrating the proportion of patients maintaining ≥70 ETDRS letters over time was performed to assess the durability of response and complement the TIR approach.

IOP-related adverse events and additional treatments following the FAc implant were documented.

Statistical analysis was conducted utilising SAS version 9.4. Visual acuity and IOP-related outcomes were analysed at 6, 12, 24, 36, 48, and 60 months.

The Shapiro-Wilks test was used to assess the normality of quantitative variables.

Since the variables followed a normal distribution, the one-way ANOVA test was used to analyse the evolution of VA among swimlane groups.

A log-rank test was used to compare the Kaplan–Meier curves for “time to a loss of VA of ≥60 ETDRS letters” across the three baseline-VA swimlanes (0–33, 34–68, and 69–100 letters).

A two-tailed paired Student’s t-test was employed to evaluate differences between the baseline visit and subsequent follow-up visits within each swimlane group and to explore differences when classified by duration of DMO (short-term and long-term) and by swimlane group.

Categorical variables were compared using a Chi squared test. *P* values of less than 0.05 were considered significant.

## Results

### Baseline demographic and clinical characteristics

The study included 671 eyes from 542 patients, with a mean ± SD age of 75.8 ± 11.0 years, and 295 (44.0%) were female. A total of 322 (48.0%) eyes were classified as “short-term” chronic DMO. The mean VA at baseline was 52.1 ± 19.3 letters. At baseline, 553 (82.4%) were pseudophakic, and 109 (16.2%) were phakic. The mean follow-up duration was 33.8 ± 14.8 months, with 419 (62.4%) eyes followed for ≥36 months. Baseline demographics are detailed in Table S2.

### Visual acuity

#### Overall

In the overall study population, VA improved from 52.1 ± 19.3 letters at baseline to 55.3 ± 20.9 letters at the 36-month visit (mean change: +3.2 ± 19.9 letters; *p* = 0.033).

#### Swimlane analysis

**Fig. 1:**
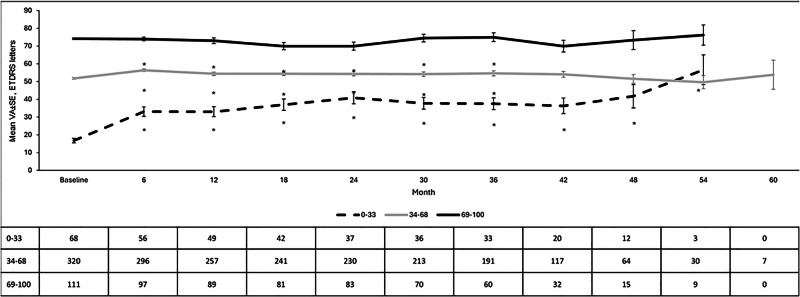
Mean visual acuity (VA) by swimlane. Vertical bars represent standard error of the mean (SE).Significant differences were observed between the three study groups at all follow-up points measured (*p* < 0.05, ANOVA test). As compared to baseline, VA improved significantly in all the swimlane groups (*p* < 0.05 each, respectively).

VA improved significantly in all swimlane groups with statistically significant differences in the magnitude of the change (Fig. 1).

**Fig. 2:**
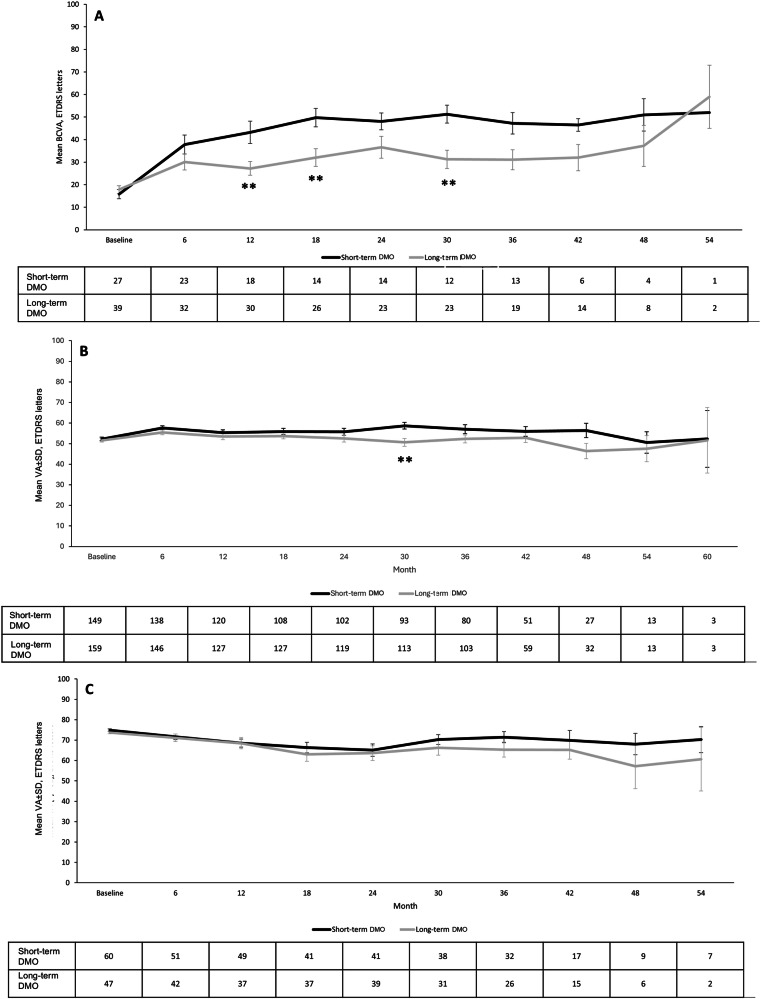
Overview of the mean visual acuity (VA) by swimlane in the eyes with short-term and long-term chronic diabetic macular oedema (DMO). Vertical bars represent standard deviation.**A** Baseline VA 0-33 ETDRS letters. **B** Baseline VA 34-68 ETDRS letters. **C** Baseline VA 69-100 ETDRS letters. **p < 0.01 between groups.

On stratification by duration of DMO (Fig. 2A), differences in VA improvements between short- and long-term groups were observed. The difference in the gain in VA was significantly greater in the short-term compared to the long-term group at most time points (at 12-, 18-, and 30-months *p* = 0.005, *p* = 0.006, and *p* = 0.003, respectively) in the lowest baseline VA category of 0 to 33 letters. In the mid baseline VA category (34 to 68 letters) the difference in gain by duration of DMO was significantly greater in the short-term group only at month 30 (*p* = 0.002).

In the best baseline VA group ≥69 letters (Fig. 2C), no significant differences in change in VA were observed between the short-term and long-term DMO groups throughout the follow-up period.

The proportion of eyes that achieved VA improvements of ≥5, ≥10, and ≥15 letters, as well as that in which VA remained stable or declined by the 36-month visit after FAc implantation is shown in Table 1.

**Table 1:** A comparison, categorised by baseline visual acuity (VA) swimlane, of the proportion of eyes who exhibited improvement, decline, or stability their best-corrected visual acuity as compared to baseline.

VA change from baseline to month 36	Baseline VA (ETDRS letters)			p^a^
	0 – 33 (*n* = 33)	34 – 68 (*n* = 191)	69 – 100 (*n* = 60)	
≥5 letter gain, %	78.8	45.5	28.3	<0.0001
≥10 letter gain, %	63.6	29.8	10.0	<0.0001
≥15 letter gain, %	48.5	19.4	5.0	<0.0001
VA stable (±4 letters), %	6.1	24.1	25.0	0.0595
≥5 letter loss, %	15.2	26.7	35.0	0.1168
≥10 letter loss, %	6.1	17.3	28.3	0.0238
≥15 letter loss, %	6.1	12.0	21.7	0.0680
Stable / improved BCVA, %	84.8	71.7	60.0	0.0367
Proportion achieving ≥6/12 vision, %	6.1	20.9	53.3	<0.0001

^a^Chi-squared test.

n Number of eyes with data at month 36.

*VA* Visual acuity, *ETDRS* Early Treatment Diabetic Retinopathy Study.

At the 36-month visit, 84.8%, 71.7%, and 60.0% of eyes in the baseline VA categories of 0–33, 34–68, and 69–100 letters, demonstrated maintained or improved VA compared to baseline values (*p* = 0.0367, Chi-squared test) (Table 1).

Table S3 presents the proportion of eyes, sub-classified both by baseline VA and duration of DMO, that demonstrated improvement, decline, or stability in their best-corrected visual acuity relative to baseline.

At the 36-month after FAc implant injection, 74 (26.1%) eyes in the total study cohort achieved a VA of 6/12 or better. When categorised by swimlane, 2 (6.1%) eyes from the 0-33 letter group, 40 eyes (20.9%) from the 34-68 letter group, and 32 (53.3%) eyes from the 69-100 letter group achieved a VA of 6/12 or greater (see Table 1, Table S3 and Fig. S1).

### Time-In-range

#### Time-in-range by visual acuity swimlane

The mean time-in-range for the FAc implant, based on three VA thresholds, without adjustment for baseline VA, is shown in Fig. S2.

Using a VA letter score threshold of ≥70 (equivalent to 6/12 or better), the mean time for study eyes to be remain within this range was significantly longer, in the >69 ETDRS letters subgroup (892.7 ± 413.4 days) compared to the mid VA group of 34–68 ETDRS letters (648.4 ± 366.4 days) and the lowest VA group 0-33 ETDRS letters achieving around 251.3 ± 175.9 days; *p* < 0.00001 (ANOVA test).

**Fig. 3:**
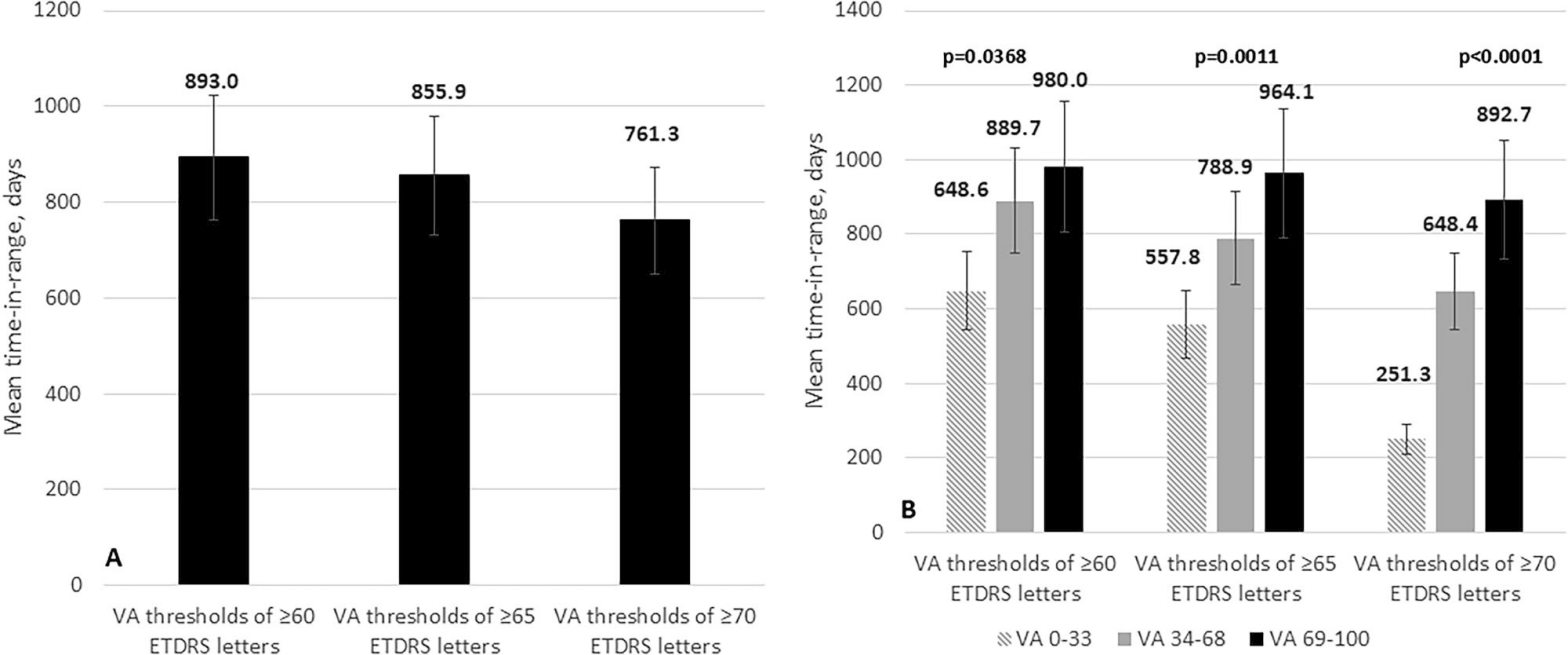
Mean time-in-range for intravitreal fluocinolone acetonide (FAc) implant. **A** Stratified according to VA letter score thresholds of ≥60 ETDRS letters, ≥65 ETDRS letters and ≥70 ETDRS letters, stratified by baseline VA siwmlanes. Vertical bars indicate standard deviation. Statistical significance was determined using a one-way ANOVA test. VA: Visual acuity. **B** The Kaplan–Meier estimates of the proportion of eyes maintaining ≥70 ETDRS letters over 60 months, stratified by baseline VA swimlane (0–33, 34–68, and 69–100 letters). In the 69–100 letters group, the estimated probability rises rapidly after implantation: by approximately 24 months, around 50% of eyes are maintaining ≥70 letters, and by 60 months, nearly 100% of these eyes reach and remain at that threshold. Eyes in the 34–68 letters swimlane show a slower, more gradual increase. Fewer of these eyes ever attain ≥70 letters, but the probability still climbs steadily over time, reaching roughly 30–40% by Year 5. Eyes in the 0–33 letters swimlane rarely achieve ≥70 letters: the red curve stays near zero for almost the entire follow-up, indicating that very few eyes with such low baseline VA ever reach the 70-letter threshold.

On selecting a VA letter score threshold of ≥65 letters, the mean TIR was significantly longer in the higher VA subgroup (964.1 ± 40.3 days) than in the other two; namely 34-68 ETDRS letters (788.9 ± 414.8 days) and the 0-33 ETDRS (557.8 ± 422.7 days) (*p* = 0.0011; ANOVA test) respectively. In eyes with VA letter score ≥60, the subgroup with baseline VA > 69 letters had a significantly longer median TIR (980.0 ± 389.5 days) than the others 34-68 ETDRS letters subgroup (889.7 ± 433.5) and the 0-33 ETDRS letters subgroup (648.6 ± 424.4 days); *p* = 0.0368 (ANOVA test) (Fig. 3A).

Additionally, the Kaplan–Meier plot clearly demonstrates that higher baseline VA is strongly associated with both a greater and faster chance of reaching and maintaining ≥70 letters after FAc implantation. Eyes starting in the 69–100 swimlane not only achieve the 70-letter mark much sooner, but almost all of them sustain it long term, whereas eyes with baseline VA <34 letters almost never reach that level (Fig. 3B).

Bootstrap resampling (2000 iterations), showed that mean TIR (weeks) increased with higher baseline VA swimlane. For the group with BCVA ≥70, mean TIR was 127.5 weeks (95% CI: 113.6–140.6; *n* = 80) in the 69–100 swimlane, compared with 92.6 weeks (95% CI: 80.8–104.6; *n* = 75) in the 34–68 swimlane, and 35.9 weeks (95% CI: 7.0–52.6; *n* = 3) in the 0–33 swimlane (Table S4 and Fig. S3).

#### Time-in-range by DMO duration

**Fig. 4:**
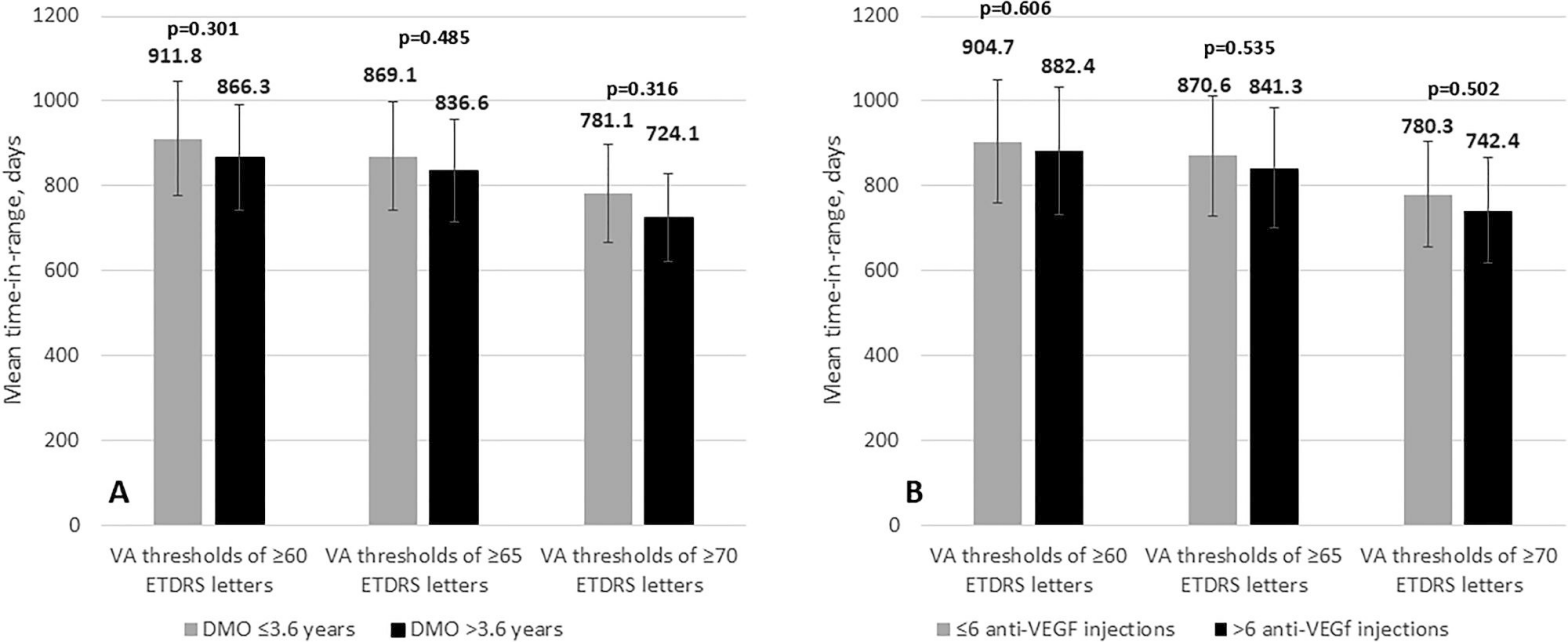
Mean time-in-range for intravitreal fluocinolone acetonide (FAc) implant. **A** Time-in-range is shown for VA thresholds of ≥60 ETDRS letters, ≥65 ETDRS letters, and ≥70 ETDRS letters, categorised by the duration of DMO. Error bars represent standard deviation. Statistical significance was assessed using a two-way Student’s t-test. **B** Time-in-range is shown for the same VA thresholds (≥60, ≥65, and ≥70 ETDRS letters), stratified by the number of prior anti-VEGF injections. Error bars indicate standard deviation, and statistical significance was evaluated using a two-way Student’s t-test. VA Visual acuity, DMO Diabetic macular oedema.

When evaluating time-in-range as a function of DMO duration, no significant differences were observed, irrespective of the selected VA letter score threshold (Fig. 4A).

Using a VA letter score threshold of ≥70 (equivalent to 6/12 or better), the mean duration that study eyes remained within this range was comparable between eyes with DMO duration ≤3.6 years (911.8 ± 433.0 days) and those with DMO duration >3.6 years (866.3 ± 415.6 days), with no statistically significant difference (*p* = 0.301). Similarly, no significant differences were observed when applying a VA letter score threshold of ≥65 letters (869.1 ± 420.7 days vs. 836.6 ± 397.5 days, *p* = 0.485) or ≥60 letters (781.1 ± 414.4 days vs. 724.1 ± 397.2 days, *p* = 0.316) (Fig. 4A).

Pairwise comparisons with adjustment (Benjamini–Hochberg procedure) for multiple testing confirmed significant differences between the 34–68 and 69–100 swimlanes for BCVA thresholds of ≥65 (adjusted *p* = 0.0026) and ≥70 (adjusted *p* = 0.0009). Findings did not change in a sensitivity analysis using multiple imputation (20 imputations) and when duration of DMO was modelled by median split and tertiles (Table S5).

#### Time-in-range by number of previous Anti-VEGF treatment

Upon stratification based on the number of prior anti-VEGF injections (≤ 6 injections versus >6 injections), no significant differences were observed, regardless of the visual acuity (VA) letter score thresholds applied (Fig. 4B).

### IOP-related adverse events

Mean intraocular pressure (IOP) increased across swimlane groups but remained similar in magnitude (*p* > 0.05, ANOVA) (Fig. S3). IOP-related adverse events were generally transient and managed with topical ocular-hypotensive medication. Laser trabeculoplasty was required in 10 (1.4%) eyes, and trabeculectomy in 14 (2.0%) eyes.

### Post-FAc implant DMO treatments

During follow-up, 253 (50.7%) eyes required additional DMO treatments, with no significant differences between VA swimlane groups (*p* = 0.1050, ANOVA). The distribution of post-FAc implant treatments by VA swimlane and DMO duration is detailed in Table S6.

## Discussion

This post-hoc analysis of the IRISS study evaluated the evolution of VA over time following administration of the 0.19 mg FAc implant in eyes with DMO, stratified by baseline VA and disease duration. Additionally, the study explored TIR as a clinically relevant functional endpoint to assess treatment response.

### Visual acuity

Among 671 eyes with suboptimal response to prior therapies, FAc implant treatment significantly improved VA, particularly in eyes with shorter DMO duration and worse initial VA. Notably, 71.7% and 60.0% of eyes with baseline VA of 34–68 and 69–100 ETDRS letters, respectively, maintained, or improved VA at 36 months. These results align with randomised trials [16–18] and real-world studies [21, 26–32], confirming that lower baseline VA correlates with significant post-treatment gains [16–19].

The present study confirmed current evidence, demonstrating that eyes in the lowest baseline VA category experience significant improvements in visual acuity following treatment [16–19].

An important finding from the present analysis was the effect of disease duration as a critical factor influencing functional outcomes. Specifically, among eyes with a baseline VA of 0-33 letters, the proportion of eyes achieving an improvement in VA of ≥5, ≥10, or ≥15 letters were significantly greater in those with short-term disease duration (≤3.6 years from diagnosis) compared to those with long-term disease duration (>3.6 years from diagnosis). This observation, coupled with the higher proportion of eyes with visual acuities between 34–68 and 69–100 that achieved or maintained a specified vision threshold throughout the study follow-up, underscores the advantages of initiating treatment with FAc implants prior to the onset of irreversible damage to the macular tissues.

These findings align with the results of the PALADIN study, in which earlier use of the implant during the treatment course resulted in better outcomes [31] in patients with DMO.

#### Time-in-range

TIR analysis, which assesses the duration eyes remained within defined VA thresholds, offers insight into functional vision stability over time. This approach is clinically relevant as it correlates with daily activities such as driving and reading [33, 34].

In clinical trials, a score of 70 ETDRS letters or 6/12 is typically regarded as a threshold for good functional vision [35]. In this study, eyes with baseline VA of 69–100 letters exhibited prolonged TIR, supporting the efficacy of FAc implants in maintaining functional vision.

While TIR has previously been employed as a metric for monitoring treatment efficacy in diabetic patients by assessing blood glucose control [22], to our knowledge, only two other studies have explored the use of TIR for evaluating treatment outcomes in eyes with DMO [23, 33]. Additionally, there is data from a post-hoc analysis that examined the duration of vision loss of ≥5, ≥10, and ≥15 letters or visual acuity worse than 20/40, by calculating patient-weeks of vision impairment in individuals with non-proliferative diabetic retinopathy. This analysis employed an inverse approach, focusing on “time out of range” [36].

The IRISS data utilised to investigate the TIR concept serves as a framework for illustrating the application of this analytical method. It highlights how TIR evaluates the consistency of treatment efficacy over time above predefined thresholds, rather than relying on a static and a single-point measurement at the trial’s conclusion. TIR could offer physicians an alternative or complementary approach when discussing clinical trial outcomes with patients, by quantifying the number of weeks a patient might expect to maintain visual acuity above a specific threshold under a given treatment.

This study has several limitations. First, it was a post-hoc analysis, Second, the method used to calculate TIR presents challenges, particularly when applied to a progressive disease with continuous outcomes like DMO. Third, VA was not assessed at all follow up visits during routine care and also visit intervals were not evenly spaced unlike clinical trials. Fourth, the analysis also assumes that study eyes had the same VA value until the next measurement was obtained. Nonetheless all available VA measurements were utilised and we believe that the TIR values we obtained are more likely to align better with clinical outcomes in routine care settings. Fifth, the sensitivity analysis for missing data employed an iterative multivariate imputer as a pragmatic check rather than a fully specified multiple imputation by chained equations (MICE) model. An additional limitation of this study is that short- and long-term DMO duration was defined based on cohort characteristics rather than the 3-year threshold used in the FAME trials [16–18]. Although duration was determined from documented clinical diagnosis, which strengthens the reliability of this stratification within our cohort, this might limit the generalisability of the results to other populations.

In summary, eyes with higher initial VA maintained superior VA outcomes throughout the 36-month therapeutic duration of the FAc implant. Additionally, eyes presenting with lower initial VA and a shorter duration of DMO at the onset of FAc treatment demonstrated significant visual acuity gains. TIR represents a clinically relevant endpoint for evaluating functional treatment outcomes longitudinally, has provided a more comprehensive assessment of functional benefit with the FAc implant and is of greater relevance compared to isolated VA measures commonly used in DMO studies.

## Summary

### What was known before

- The ILUVIEN Registry Safety Study (IRISS) is the longest and largest real-world study to date on the outcomes of intravitreal fluocinolone acetonide (FAc) implantation in European clinical practice with clinically meaningful improvements in visual acuity (VA) and no new or unexpected safety signals.
- IRISS confirmed the favourable, long-term benefit-to-risk profile of the FAc implant in eyes with chronic DMO, with an additional benefit in patients when this therapy is administered earlier.

### What this study adds

- When stratified based on baseline visual acuity, the visual improvements were particularly evident in eyes with worse VA at baseline and a shorter duration of DMO prior to treatment.
- The improved vision in all subgroups has been stable up to 36 months following implantation of the FAc implant.
- Time-in-Range analysis demonstrated that eyes with better baseline visual acuity, when treated with the FAc implant, maintain a vision of 6/12 or better for longer periods.

## Supplementary information

Supplementary Annex I and Tables/figures

## Data Availability

The datasets used and/or analysed during the current study are available from the corresponding author on reasonable request.
