# Supplementary material for: Impact of baseline visual acuity, time-in-range and early treatment on functional outcomes in DMO: insights from the IRISS outcomes
Source: Eye (Lond). 2025 Dec 3;40(2):252–9. doi: 10.1038/s41433-025-04102-8 (PMC12830729; doi:10.1038/s41433-025-04102-8)
Supplement: Supplementary file 1 — Supplementary Annex I and Tables/figures [file 41433_2025_4102_MOESM1_ESM.docx]

**SUPPLEMENTARY TABLES/FIGURES**

**Table S1. Participating Members of the ILUVIEN Registry Safety Study (IRISS).**

| **Name** | **Affiliation** |
| --- | --- |
| Robin Hamilton | NIHR Moorfields Clinical Research Facility, London |
| Usha Chakravarthy | The Queen’s University and Royal Group of Hospitals Trust, Belfast |
| Guzaliya Safiullina | Gloucestershire Hospitals NHS, Cheltenham |
| Geeta Menon | Unit Frimley Park Hospital, Surrey |
| Ramesh Sivaraj | Heart of England NHS Foundation Trust, Birmingham |
| Haralabos Eleftheriadis | Laser and Retinal Research Unit, King’s Health Partners, London |
| Simon Taylor | Royal Surrey County Hospital, Guildford |
| Saad Younis | ICORG - Imperial College, London |
| Konstantinos Balaskas | Manchester Royal Eye Hospital |
| Vasant Raman | Derriford Hospital, Plymouth, Devon |
| Fahd Quhill | Royal Hallamshire Hospital, Sheffield Teaching Hospitals NHS |
| Bushra Mushtaq | Sandwell and West Birmingham Hospital NHS Trust |
| Nicholas Glover | Queen Elizabeth Hospital, Birmingham |
| Ben Burton | James Paget University Hospital, Gorleston |
| David Steel | Sunderland Royal Hospital, Sunderland |
| Salim Natha | Wrightington, Wigan and Leigh Eye Unit, WWL NHS Trust, Wigan |
| Somnath Banerjee | University Hospital Leicester NHS, Leicester |
| Deepthi Seneviratne | Leighton Hospital, Crewe |
| Nishal Patel | Kent and Canterbury Hospital, East Kent Hospitals University, Canterbury |
| Spyridon Mourtzoukos | Queen Alexandra Hospital, Portsmouth Hospitals NHS |
| Riaz Asaria | Royal Free Hospital, London |
| Ajay Bhatnagar | New Cross Hospital, The Royal Wolverhampton NHS |
| Rehna Khan | Calderdale Hospital Eye Clinic, Calderdale and Huddersfield NHS Trust |
| Ahmed Kamal | Aintree University Hospital, Liverpool |
| Clare Bailey | Bristol Eye Hospital, University Hospitals Bristol |
| Louise Downey | Hull & East Yorkshire Eye Hospital, Hull |
| Anil Kumar | Queen's Hospital, Burton Hospitals NHS, Burton-on-Trent |
| Gonçalo Almeida | Maidstone Hospital, Maidstone, Kent |
| Patrick Richardson | Royal Derby Hospital, Derby |
| Hadi Zambarakji | Whipps Cross University Hospital, London |
| Colin Jones | Norfolk and Norwich University Hospital, Norwich |
| Katrin Lorenz | University Medical Center, Johannes Gutenberg-University Mainz |
| Karl U. Bartz-Schmidt | University Hospital Tuebingen, Tuebingen |
| Frank Holz | Department of Ophthalmology, University of Bonn |
| Lars Wagenfeld | University Medical Center Hamburg-Eppendorf, Hamburg |
| Peter Wiedemann | University Eye Hospital Leipzig, Leipzig |
| Rainer Guthoff | University of Düsseldorf |
| Manfred Tetz | Eye Centre Spreebogen, Berlin |
| Gerd U. Auffarth | International Vision Correction Research Centre, University of Heidelberg |
| Christian Ksinsik | Gemeinschaftspraxis am Glacis, Torgau, Germany |
| Frank Koch | Universitätsklinikum Frankfurt, Klinik für Augenheilkunde |
| Helmut Sachs | Krankenhaus Dresden-Friedrichstadt |
| Dirk Sandner | Universitätsklinikum Carl Gustav Carus, Klinik für Augenheilkunde |
| Berthold Seitz | Universitätsklinikum des Saarlandes, Augenklinik |
| João Figueira | AIBILI, Coimbra |
| Ângela Carneiro | Department of Ophthalmology, Porto Medical School / Hospital S. João, Porto |
| José Henriques | Instituto de Retina e Diabetes Ocular de Lisboa (IRL), Lisbon |
| Rufino Silva | Espaço Médico de Coimbra, Coimbra |
| Miguel Amaro | Hospital de Vila Franca de Xira, Vila Franca de Xira |
| Angelina Meireles | Hospital de Santo António, Porto |
| João Paulo Castro Sousa | Centro Hospitalar de Leiria, Leiria |
| António Rodrigues | Centro Hospitalar de Lisboa Ocidental, EPE |
| Rui Carvalho | Hospital Pedro Hispano (Unidade Local de Sáude de Matosinhos, EPE) |

**Annex I.**

**Definition and calculation of TIR**
TIR for a given BCVA threshold *L* (≥60, ≥65, ≥70 ETDRS letters) was defined as the cumulative duration (in weeks) during which an eye’s recorded BCVA was at or above *L*. Formally, let tit_iti​ denote the interval between visits iii and i+1i+1i+1, and ViV_iVi​ the recorded BCVA at visit iii; then, for a threshold LLL, TIR is defined as:

$$TIR(L)=a_{0}+\sum_{i=1}^{n-1} ti.1\left( Vi\geq L \right)$$

where 1(⋅) is the indicator function. This assumes that BCVA remains constant between visits. We acknowledge that this may introduce bias under irregular follow-up, but this is consistent with standard practice in real-world ophthalmology datasets and provides an interpretable summary of “time spent above threshold” that is clinically meaningful.

**Justification of thresholds**
The thresholds (BCVA ≥60, ≥65, and ≥70 letters) were chosen to reflect clinically relevant functional cut-offs corresponding to levels of visual performance and treatment targets commonly used in clinical trials and practice. Reporting results across three thresholds provides transparency and allows readers to assess robustness across different definitions of preserved vision.

**Quantifying uncertainty and robustness**

- **Uncertainty:** We estimated 95% bootstrap percentile confidence intervals (2,000 resamples) for mean TIR by threshold and baseline VA swimlane. These results are reported in Supplementary Table S4 and illustrated in Supplementary Figure S2. Across all thresholds, eyes in higher baseline swimlanes demonstrated substantially longer mean TIR.
- **Multiple testing:** To test group differences, we performed Mann–Whitney U tests for all pairwise comparisons and controlled for multiple testing using the Benjamini–Hochberg false discovery rate procedure. The difference between the 34–68 and 69–100 swimlanes remained significant for BCVA ≥65 (*p* = 0.0026, adjusted) and ≥70 (*p* = 0.0009, adjusted) (Supplementary Table S5).
- **Missing data sensitivity:** We performed multivariate iterative imputation (20 imputations) using predictors including baseline swimlane, DMO duration, and other TIR variables. Imputed means were consistent with observed means, suggesting that findings are not driven by missing values. We explicitly note in the revised manuscript that missingness is greater at higher thresholds, particularly in subgroups with few eyes achieving ≥70 letters, which reduces precision.
- **Interpolation sensitivity:** To further evaluate the assumption of constant BCVA between visits, we performed a pragmatic sensitivity analysis using alternative definitions of exposure time (based on median split and tertiles of DMO duration). Results were not materially different from the primary analysis, supporting the robustness of our findings.

**Table S2. Main demographic and clinical characteristics of the study population.**

| **Variable** | **N=671** |
| --- | --- |
| Age, years  Mean±SD | 75.8±11.0 |
| Sex, n (%)  Women  Men | 295 (44.0)  376 (56.0) |
| Duration DMO, years  Mean±SD | 4.5±3.9 |
| Lens status, n (%)  Aphakic  Phakic  Pseudophakic  N.A. | 5 (0.7)  109 (16.2)  553 (82.4)  4 (0.6) |
| FAc implant, n (%)  Unilateral  Bilateral | 413 (61.6)  258 (38.4) |
| VA, letters^⁂^  Mean±SD | 52.1±19.3 |
| Distribution of eyes by swimlane, n (%)  0-33  34-68  69-100  Missing data | 68 (10.1)  320 (47.7)  111 (16.5)  172 (25.6) |
| IOP, mmHg  Mean±SD | 15.4±3.2 |

*Total amount may be greater than 100%.

**Not specified.

^†^Percentages were calculated based on the eyes that underwent laser treatment.

^⁂^Early Treatment Diabetic Retinopathy Study (ETDRS) letters.

^1^Missing data in 172 eyes,

SD: Standard deviation; DMO: Diabetic macular oedema; NA: Not available; PRP: Pan-Retinal Photocoagulation; Anti-VEGF: Vascular endothelial growth factor inhibitors; SAC-i: Short-acting corticosteroids intravitreal implant; IVC: Intravitreal corticosteroids injections; VA: Visual acuity.

**Table S3. A comparison, stratified by** **baseline visual acuity (VA) swimlane and diabetic macular oedema (DMO) duration, of the proportion of eyes that displayed improvement, decline, or stability in their best corrected visual acuity in relation to baseline.**

| **VA change from baseline**  **to month 36** | **Baseline VA (ETDRS letters)** | | | | | | | | |
| --- | --- | --- | --- | --- | --- | --- | --- | --- | --- |
|  | **0 – 33** | | | **34-68** | | | **69-100** | | |
|  | Short-term  (n=13) | Long-term  (n=19) | p^a^ | Short-term  (n=80) | Long-term  (n=103) | p^a^ | Short-term  (n=32) | Long-term  (n=26) | p^a^ |
| ≥5 letter gain, % | 100.0 | 63.2 | 0.0252 | 47.5 | 41.7 | 0.4566 | 28.1 | 26.9 | 1 |
| ≥10 letter gain, % | 92.3 | 42.1 | 0.0079 | 32.5 | 26.2 | 0.4121 | 6.3 | 15.4 | 0.3926 |
| ≥15 letter gain, % | 76.9 | 31.6 | 0.0290 | 22.5 | 15.5 | 0.2541 | 0.0 | 11.5 | 0.0843 |
| VA stable (±4 letters), % | 0.0 | 10.5 | 0.5020 | 28.8 | 22.3 | 0.3908 | 34.4 | 15.4 | 0.1359 |
| ≥5 letter loss, % | 0.0 | 26.3 | 0.0641 | 21.3 | 32.0 | 0.1324 | 25.0 | 46.2 | 0.1055 |
| ≥10 letter loss, % | 0.0 | 10.5 | 0.5020 | 15.0 | 20.4 | 0.4389 | 18.8 | 38.5 | 0.1403 |
| ≥15 letter loss, % | 0.0 | 10.5 | 0.5020 | 10.0 | 14.6 | 0.3799 | 12.5 | 30.8 | 0.1111 |
| Stable / improved BCVA, % | 100.0 | 73.7 | 0.1000 | 77.5 | 66.0 | 0.1000 | 71.9 | 46.2 | 0.1000 |
| Proportion achieving ≥6/12 vision,% | 15.4 | 0.0 | 0.1573 | 25.0 | 17.5 | 0.2705 | 62.5 | 42.3 | 0.1860 |

^a^Fisher's exact test

n=Number of eyes with data at month-36.

VA: Visual acuity; ETDRS: Early Treatment Diabetic Retinopathy Study.

**Table S4. Bootstrap (2,000 resamples) mean Time-in-Range (TIR) in weeks with 95% percentile confidence intervals, stratified by baseline visual acuity (VA) swimlane and best corrected visual acuity (BCVA) threshold (≥60, ≥65, ≥70 ETDRS letters).**

Time-in-Range (TIR) for a given BCVA threshold *L* was computed as the cumulative duration (in weeks) that an eye’s recorded BCVA was at or above *L*. For transparency, we report TIR for three thresholds (BCVA ≥ 60, ≥ 65 and ≥ 70 letters). To quantify uncertainty, we computed 95% bootstrap percentile confidence intervals (based on 2,000 resamples) for mean TIR estimates by baseline swimlane.

| **Threshold label** | **Swimlane** | **n** | **Mean (95%CI)** |
| --- | --- | --- | --- |
| **BCVA ≥ 60 letters** | 0-33 | 9 | 92.65 (55.48-129.21) |
|  | 34-68 | 194 | 127.10 (118.84-135.62 |
|  | 69-100 | 98 | 140.00 (129.11-150.84) |
| **BCVA ≥ 65 letters** | 0-33 | 6 | 79.69 (38.61-122.62) |
|  | 34-68 | 157 | 112.70 (103.27-121.50) |
|  | 69-100 | 96 | 137.73 (125.81-148.52) |
| **BCVA ≥ 70 letters** | 0-33 | 3 | 35.90 (7.00-52.57) |
|  | 34-68 | 75 | 92.63 (80.79-104.62) |
|  | 69-100 | 80 | 127.53 (113.63-140.57) |

N: Number of non-missing observations included in each estimate.

**Table S5. Pairwise comparisons of Time-in-Range (TIR, weeks) between baseline visual acuity (VA) swimlanes within each best corrected visual acuity (BCVA) threshold.**
To evaluate robustness to missing data and interpolation assumptions, a sensitivity analysis was conducted using multivariate iterative imputation (20 imputations), followed by repeated group comparisons; results were consistent with the primary analysis. Pairwise group comparisons were performed using Mann–Whitney U tests, with *p*-values adjusted for multiple testing by the Benjamini–Hochberg false discovery rate procedure.

| **Threshold label** | **Short-term DMO^1^** | **Long-term DMO^2^** | **p^a^** | **p^b^** |
| --- | --- | --- | --- | --- |
| **BCVA ≥ 60^c^** | 0-33 | 34-68 | 0.1291 | 0.1452 |
|  | 0-33 | 69-100 | 0.0316 | 0.0569 |
|  | 34-68 | 69-100 | 0.0789 | 0.1015 |
| **BCVA ≥ 65^c^** | 0-33 | 34-68 | 0.1982 | 0.1982 |
|  | 0-33 | 69-100 | 0.0296 | 0.0569 |
|  | 34-68 | 69-100 | 0.0006 | 0.0026 |
| **BCVA ≥ 70^c^** | 0-33 | 34-68 | 0.0454 | 0.0681 |
|  | 0-33 | 69-100 | 0.0211 | 0.0569 |
|  | 34-68 | 69-100 | <0.0001 | 0.0009 |

^a^Mann–Whitney U test p-values.

^b^Benjamini–Hochberg false-discovery-rate adjusted p-values across all pairwise tests reported.

^c^ETDRS Letters.

^1^Duration of DMO ≤3.6 years.

^2^Duration of DMO >3.6 years.

DMO: Diabetic macular oedema; BCVA: Best corrected visual acuity.

**Table S6. Summary of post intravitreal fluocinolone acetonide (FAc) implant diabetic macular oedema (DMO) adjuvant treatments categorized by VA swimlanes and stratified by the duration of diabetic macular oedema (DMO).**

| **Post FAc implant treatments** | **Baseline VA (ETDRS letters)** | | | | | | | | | | |
| --- | --- | --- | --- | --- | --- | --- | --- | --- | --- | --- | --- |
|  | **0 – 33** (n=68) | | | **34-68** (n=320) | | | **69-100** (n=111) | | | **P^1^** | **P^2^** |
|  | Short-term  (n=27) | Long-term  (n=39) | p | Short-term  (n=149) | Long-term  (n=159) | p | Short-term  (n=60) | Long-term  (n=47) | p |  |  |
| Any Treatments for DMO  Patients, n (%)  Mean number of treatments | 10 (37.0)  6.4±8.0 | 17 (43.6)  7.9±8.9 | 0.6211^a^  0.6570^c^ | 83 (55.7)  10.6±11.4 | 76 (47.8)  11.9±12.5 | 0.1729^a^  0.4980^c^ | 34 (56.7)  8.9±7.1 | 25 (53.2)  6.8±7.5 | 0.8450^a^  0.2640^c^ | 0.1765^b^  0.3707^d^ | 0.6666^b^  0.0949^d^ |
| Intravitreal Anti-VEGF  Patients, n (%)  Mean±SD number of treatments | 6 (22.2)  8.8±8.4 | 13 (33.3)  9.1±8.8 | 0.4120^a^  0.9550^c^ | 58 (38.9)  12.6±10.9 | 60 (37.7)  12.6±12.3 | 0.9067^a^  0.9820^c^ | 24 (40.0)  11.0±6.0 | 16 (34.0)  6.0±7.5 | 0.5525^a^  0.0250^c^ | 0.2253^b^  0.5764^d^ | 0.8214^b^  0.0951^d^ |
| Intravitreal Steroid  Patients, n (%)  Mean number of treatments | 3 (11.1)  2.3±0.6 | 3 (7.7)  2.0±1.7 | 0.6823^a^  0.7680^c^ | 34 (22.8)  3.0±3.2 | 27 (17.0)  4.2±4.8 | 0.2523^a^  0.2400^c^ | 12 (20.0)  1.6±0.9 | 8 (17.8)  5.9±6.8 | 0.8048^a^  0.0420^c^ | 0.3804^b^  0.3285^d^ | 0.3390^b^  0.5129^d^ |
| Laser*  Patients, n (%)  Mean number of treatments | 2 (7.4)  2.0±0.0 | 5 (12.8)  2.2±1.3 | 0.6911^a^  0.8460^c^ | 28 (18.8)  1.8±1.5 | 19 (11.9)  1.7±0.9 | 0.1132^a^  0.8250^c^ | 12 (20.0)  1.8±1.7 | 12 (25.5)  2.2±1.3 | 0.6411^a^  0.5130^c^ | 0.3179^b^  0.9752^d^ | 0.0649^b^  0.4970^d^ |
| Vitrectomy  Patients, n (%)  Mean number of treatments | 0 (0.0)  NA | 1 (2.6)  1.0±0.0 | 1.000^a^  NA | 4 (2.7)  1.0±0.0 | 3 (1.9)  1.0±0.0 | 0.7156^a^  NA | 2 (3.3)  1.0±0.0 | 1 (2.1)  1.0±0.0 | 1.000^a^  NA | 0.6479^b^  NA | 0.9637^b^  NA |
| Time to Any Treatments for DMO, days | 322.7±233.5 | 331.9±199.0 | 0.914^c^ | 376.9±309.2 | 362.8±293.0 | 0.385^c^ | 494.0±350.9 | 420.9±278.8 | 0.208^c^ | 0.1435^d^ | 0.5782^d^ |
| Time to Intravitreal Anti-VEGF, days | 382.5±234.6 | 343.7±229.8 | 0.738^c^ | 412.6±326.8 | 438.6±357.7 | 0.341^c^ | 458.0±334.5 | 503.1 ± 212.1 | 0.326^c^ | 0.8061^d^ | 0.4377^d^ |
| Time to Intravitreal Steroid, days | 729±268.7 | 553.0±0.0 | 0.687^c^ | 573.6±383.2 | 405.5±192.1 | 0.035^c^ | 689.1±255.2 | 542.1±422.3 | 0.192^c^ | 0.6093^d^ | 0.4518^d^ |
| Time to Laser*, days | 46±30.3 | 426.4±243.5 | 0.040^c^ | 440.2±409.4 | 433.8±321.3 | 0.478^c^ | 435.5±363.9 | 339.9±233.5 | 0.255^c^ | 0.2526^d^ | 0.7214^d^ |
| Time to Vitrectomy. days | NA | 890±0.0 | NA | 301.3±191.7 | 378±368.1 | 0.365^c^ | 444.5±37.5 | 1177±0.0 | 0.020^c^ | 0.3782^d^ | 0.3274^d^ |

^a^Fisher's exact test

^b^Chi-squared test.

^c^Independent samples Student t test.

^d^ANOVA test

^1^Amongst BCVA swimlane groups and short-term DMO duration.

^2^Amongst BCVA swimlane groups and long-term DMO duration

* Laser treatment includes: focal, grid and Pan-retinal photocoagulation (PRP).

n=Number of eyes with data at baseline.

FAc: Intravitreal fluocinolone acetonide; VA: Visual acuity; ETDRS: Early Treatment Diabetic Retinopathy Study; SD: Standard deviation.

**Figure S1.** Overview of the percentage of eyes with a visual acuity (VA) of ≥6/12 at baseline (grey bar) and at 36 months following intravitreal fluocinolone acetonide (FAc) implant injection (black bar), in the overall study population and stratified by baseline VA swimlanes. A significantly higher proportion of eyes achieved a VA of ≥6/12 in the overall cohort (+10.6%; 95% CI: 4.8% to 16.8%; p=0.0003), as well as in the 0-33 (+6.1%; 95% CI: 0.9% to 19.7%; p=0.0407) and 34-68 ETDRS letter groups (+20.9%; 95% CI: 15.6% to 27.2%; p<0.0001). However, in the baseline VA 69-100 group, this proportion was reduced (-16.1%; 95% CI: -30.8% to -1.0%; p=0.0372).

**
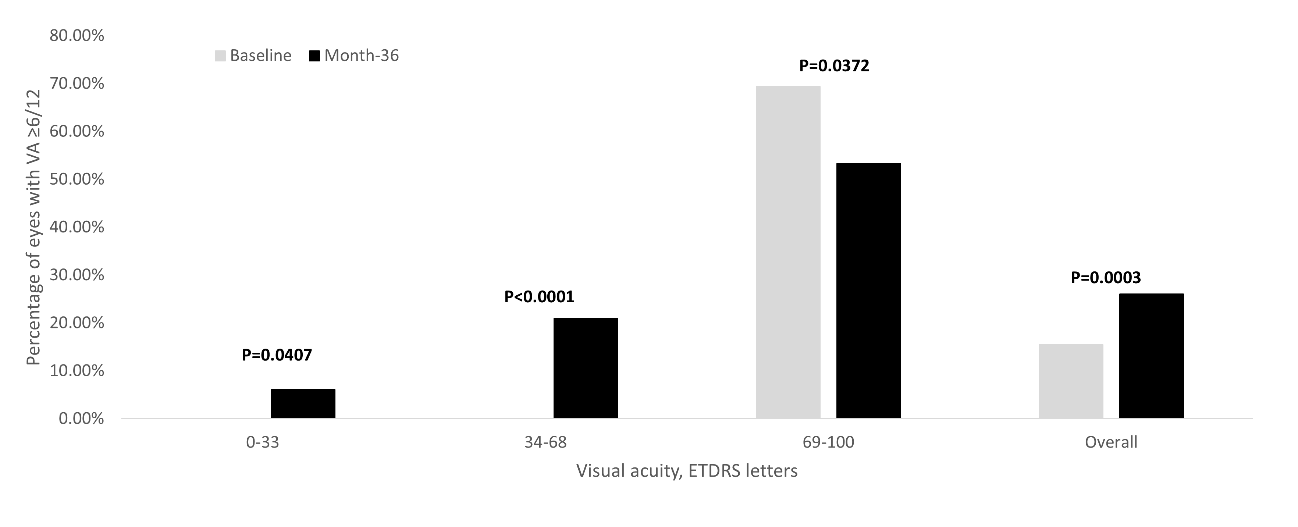
**

**Figure S2. Mean Time-in-Range (TIR) in weeks) with 95% bootstrap percentile confidence intervals by best corrected visual acuity BCVA threshold and baseline swimlane.**

**
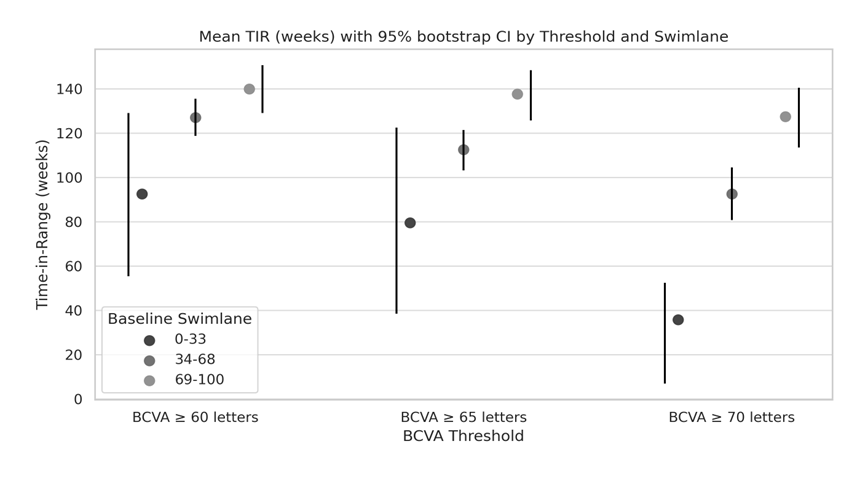
**

**Figure S3.** Mean intraocular pressure (IOP) according to baseline visual acuity (VA) swimlanes. Vertical bars represent standard deviation. No significant differences were observed among the three study groups at any follow-up time points (p>0.05, ANOVA test).

Compared to baseline, IOP was significantly greater in the 0-33 VA group at month 30 (p=0.011); in the 34-68 VA group at month 6 (p<0.0001), month 12 (p<0.0001), month 18 (p=0.0004), month 24 (p<0.0010), month 30 (p=0.0030), month 36 (p=0.012), and month 42 (p=0.022); and in the 69-100 VA group at month 6 (p<0.0001), month 12 (p<0.0001), month 18 (p<0.0001), month 24 (p<0.0001), month 30 (p=0.035), month 36 (p=0.030), and month 42 (p=0.046).

**
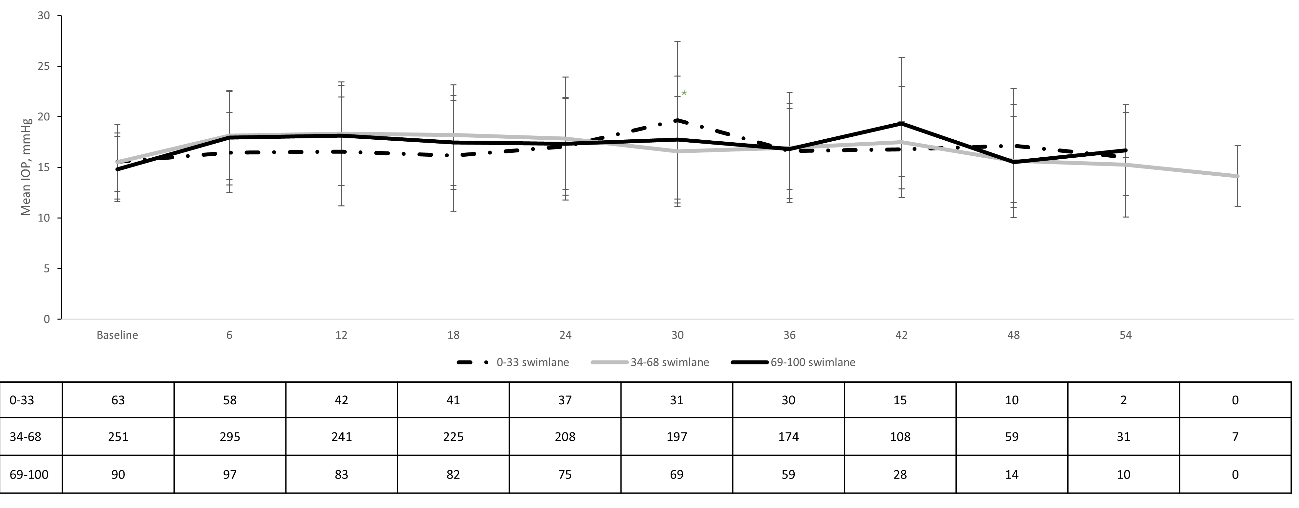
**
